# Interactions of PLA_2_-s from *Vipera lebetina*, *Vipera berus berus* and *Naja naja oxiana* Venom with Platelets, Bacterial and Cancer Cells

**DOI:** 10.3390/toxins5020203

**Published:** 2013-01-24

**Authors:** Mari Samel, Heiki Vija, Imbi Kurvet, Kai Künnis-Beres, Katrin Trummal, Juhan Subbi, Anne Kahru, Jüri Siigur

**Affiliations:** National Institute of Chemical Physics and Biophysics, Tallinn 12618, Estonia; E-Mails: mari.samel@kbfi.ee (M.S.); heiki.vija@kbfi.ee (H.V.); imbi.kurvet@kbfi.ee (I.K.); kkunnis@gmail.com (K.K.-B.); katrin.trummal@kbfi.ee (K.T.); juhan.subbi@kbfi.ee (J.S.); anne.kahru@kbfi.ee (A.K.)

**Keywords:** Snake venom, *Vipera lebetina*, *Vipera berus berus*, *Naja naja oxiana*, phospholipase A_2_, human platelet aggregation inhibition, antibacterial, bioluminescent bacteria, cancer cells

## Abstract

Secretory phospholipasesA_2_ (sPLA_2_s) form a large family of structurally related enzymes widespread in nature. Herein, we studied the inhibitory effects of sPLA_2_s from *Vipera lebetina* (VLPLA_2_), *Vipera berus berus* (VBBPLA_2_), and *Naja naja oxiana* (NNOPLA_2_) venoms on (i) human platelets, (ii) four different bacterial strains (gram-negative *Escherichia coli* and *Vibrio fischeri*; gram-positive *Staphylococcus aureus* and *Bacillus subtilis*) and (iii) five types of cancer cells (PC-3, LNCaP, MCF-7, K-562 and B16-F10) *in vitro.* sPLA_2_s inhibited collagen-induced platelet aggregation: VBBPLA_2_ IC_50_ = 0.054, VLPLA_2_ IC_50_ = 0.072, NNOPLA_2_ IC_50_ = 0.814 μM. *p*-Bromophenacylbromide-inhibited sPLA_2_ had no inhibitory action on platelets. 36.17 μM VBBPLA_2 _completely inhibited the growth of gram-positive *Bacillus subtilis* whereas no growth inhibition was observed towards gram-negative *Escherichia coli*. The inhibitory action of sPLA_2_s (~0.7 μM and ~7 μM) towards cancer cells depended on both venom and cell type. VBBPLA_2 _(7.2 μM) inhibited significantly the viability of K-562 cells and the cell death appeared apoptotic. The sPLA_2_s exhibited no inhibitory effect towards LNCaP cells and some effect (8%–20%) towards other cells. Thus, already sub-μM concentrations of sPLA_2_s inhibited collagen-induced platelet aggregation and from the current suite of studied svPLA_2_s and test cells, VBBPLA_2_ was the most growth inhibitory towards *Bacillus subtilis* and K-562 cells.

## Abbreviations

svPLA_2_: snake venom PLA_2_
sPLA_2_: secretory PLA_2_
VLPLA_2_: *Vipera lebetina* phospholipase A_2_
VBBPLA_2_: *Vipera berus berus* phospholipase A_2_
NNOPLA_2_: *Naja naja oxiana* phospholipase A_2_
*p*-BPB: *p*-bromophenacylbromide
MALDI-TOF MS: matrix assisted laser desorption ionization time-of-flight mass spectrometry
lysoPC: lysophosphatidylcholine
PRP: platelet-rich plasma

## 1. Introduction

Phospholipases A_2_ (E.C. 3.1.1.4) are enzymes that catalyze the hydrolysis of the *sn-2* fatty acyl ester bond of *sn-3* phosphoglycerides, liberating free fatty acids, and lysophospholipids. Phospholipases A_2_ (PLA_2_s) are a large family of proteins found in various mammalian tissues: arthropods, as well as in the venoms of snakes, scorpions and bees. Based on their source, catalytic activity, amino acid sequence, chain length and disulfide bond patterns, PLA_2_s are divided into 16 groups [1] including 10 groups of secretory PLA_2_s (sPLA_2_s) [2,3]. The variability of the structure of the conserved domains of sPLA_2_s from bacteria to mammals was recently investigated by Nevalainen *et al.* [4]. 

The sPLA_2_s are small-molecular-mass proteins (13–15 kDa) that require the presence of Ca^2+^ for their catalytic activity. In snake venoms, only two groups of sPLA_2_s (GI and GII) have been identified. Group I (GIA) includes the svPLA_2_s from *Elapinae* and *Hydrophiinae* venoms with 115–120 amino acid residues and these svPLA_2_s are homologous to mammalian pancreatic GIB sPLA_2_. Group II (GIIA and GIIB) comprises the svPLA_2_s from *Crotalinae* and *Viperinae* venoms with 120–125 amino acid residues and homologous to mammalian non-pancreatic Group II-A sPLA_2_ [3]. Group II PLA_2_s are in turn divided into different subgroups on the basis of amino acid residue in the 49^th^ position: catalytically active D49 enzymes, catalytically inactive or with low activity K49, S49, N49 or R49 forms [5,6]. The above described subgroups exhibit a wide variety of physiological and pathological effects. In addition to their possible role in the digestion of prey, snake venom sPLA_2_s exhibit a wide spectrum of pharmacological effects such as neurotoxicity, cardiotoxicity, myotoxicity, anticoagulant, anticancer effects *etc.* [3,5,6,7,8,9,10,11,12].

Numerous snake venom sPLA_2_s that modulate platelet function have been characterized [13,14,15,16,17,18,19] and different mechanisms of action shown [6,15,20,21,22,23,24,25,26]. The sPLA_2_s effect on platelet aggregation can be independent or dependent on their catalytic activity. However, the mechanism of action of snake sPLA_2_s on platelet aggregation is not fully elucidated. 

In addition, an increasing number of sPLA_2_s with antibacterial properties has been reported [27,28,29,30,31,32,33,34,35,36]. For example, sPLA_2_s have been shown to be inhibitory (bacteriostatic) or killing (bactericidal) to gram-positive bacteria *Staphylococcus aureus* [37]. In case of svPLA_2 _from *Crotalus durissus collilineatus* venom the bactericidal effect was entirely dependent on its enzymatic activity [38]. The effect of sPLA_2_s towards gram-positive and gram-negative bacteria and their role in the host defence against bacterial infections has been reviewed by Nevalainen *et al.* [39].

Different types of sPLA_2_s and synthetic peptides derived from sPLA_2_ homologues have been shown to possess antitumor and antiangiogenic activity against different cancer cells *in vitro.* The antitumor activities have been detected for the acidic BthA-I-PLA_2_ from *Bothrops jararacussu* venom [40], for RVV-7, a basic 7 kDa toxin from Russell’s viper venom [41], for two sPLA_2_s from *Cerastes cerastes* venom [42], for sPLA_2_ from *Naja naja atra* venom [43], for a Lys^49 ^sPLA_2_ from *Protobothrops flavoviridis* venom [44], for a Drs-PLA_2_ from *Daboia russelii siamensis* venom [45]. Recent studies have shown that MVL-PLA_2_ from *Macrovipera lebetina transmediterranea* venom inhibited cell adhesion and migration of melanoma IGR39 cells and fibrosarcoma HT1080 cells *in vitro* [46,47]. Antitumor properties of different snake venom phospholipases A_2 _have been reviewed by Rodrigues *et al.* [12].

In the current study sPLAs from *Vipera berus berus* (common viper), *Vipera lebetina* (Levantine viper) and *Naja naja oxiana* (Middle-Asian cobra) venoms were studied for their biological effects using (i) human platelets, (ii) different gram-negative (*Vibrio fischeri*, *Escherichia coli*) and gram-positive (*Bacillus subtilis*, *Staphylococcus aureus*) bacterial strains and (iii) five different cancer cells lines (prostate cancer cell lines PC-3, LNCaP, breast cancer cell line MCF-7, chronic myeloid leukemic cell line K-562 and mouse melanoma cell line B16-F10).

## 2. Results

### 2.1. Purification and Characterization of sPLA_2_s

VLPLA_2_ (*Vipera lebetina* sPLA_2_) was purified as described by Vija *et al.* [18] and VBBPLA_2_ (*Vipera berus berus* sPLA_2_) according to Križaj *et al.* [48]. In the case of NNOPLA_2_ (*Naja naja oxiana* sPLA_2_), a new two-step purification scheme involving Sephadex G-50 sf and pentylagarose chromatography was used resulting in homogeneous sample. 

The relative activity of studied svPLA_2_s was comparatively high: VLPLA_2_—882 μmol/min mg; VBBPLA_2_—1900 μmol/min mg and NNOPLA_2_—1200 μmol/min mg. The molecular masses of PLA_2_s after reduction with 2-mercaptoethanol detected by SDS-PAGE were about 14,000 Da. VLPLA_2_ had pI value in the acidic region (4.3), VBBPLA_2_ in the basic region (9.3) and NNOPLA_2_ in the neutral region (6.7). The activity of svPLA_2_ after isoelectric focusing in the gel was detected using egg-yolk overlay-technique (data not shown). 

**Figure 1:**
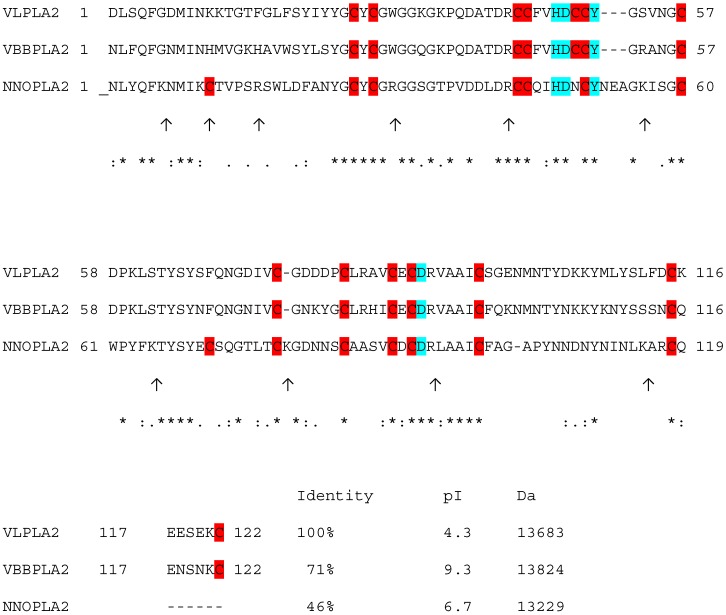
Alignment of *V. lebetina* VLPLA_2_ (EU421953) [18], VBBPLA_2_* V. berus berus* (P31854) [48] and NNOPLA_2_ isozyme E from *N. naja oxiana* (P25498) [49]. The alignment was performed using the program CLUSTAL W (1.83) multiple sequence alignment. “*” indicates positions which have a single, fully conserved residue; “:” indicates that one of the “strong” amino acid groups is fully conserved; “.” indicates that one of the “weaker” groups is fully conserved. Trypsin cleavage sites in NNOPLA_2_ are indicated as ↑. Cysteine residues are on red background, conserved catalytic network formed by four amino acid residues His48, Asp49, Tyr52 and Asp99 are on blue background.

MALDI-TOF MS analysis confirmed the molecular masses estimates of native PLA_2_s revealing single peaks for enzymes with the actual molecular masses of 13,683 Da for VLPLA_2_, 13,824 Da for VBBPLA_2_ and 13,229 Da for NNOPLA_2_. To distinguish between the possible isoforms, PLA_2_s of different venoms were subjected to trypsinolysis and the masses of the resulting peptides were analysed by MALDI-TOF MS. The peptide mass fingerprinting results confirmed that VBBPLA_2_ was a close match with enzyme formerly sequenced by Križaj *et al.* [48], VLPLA_2_ matched with sequence (EU421953) [18] and NNOPLA_2 _with enzyme isoform 3 formerly sequenced by Ovchinnikov *et al.* [49] (Figure 1). MALDI-TOF analysis of tryptic peptides derived from NNOPLA_2_ is provided in Figure S1.

### 2.2. Inhibition of Human Platelet Aggregation *in Vitro*

**Figure 2:**
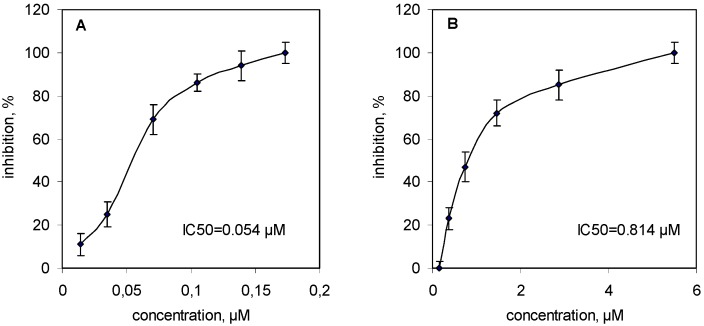
Inhibitory effects of svPLA_2_s on collagen-induced human platelet aggregation. (**A**) human platelet rich plasma (PRP) samples were stirred for 2 min at 37 °C with VBBPLA_2_s (0.014–0.173 μM) and then 2 μg/mL of collagen (final concentration in the test) was added to induce platelet aggregation; (**B**) The PRP samples were preincubated with NNOPLA_2_s (0.148–5.510 μM) under the same conditions. Results are reported as means ± SD (*n* = 3).

sPLA_2_s from all three venoms inhibited collagen-induced platelet aggregation in platelet-rich plasma in a concentration-dependent manner: the IC_50_ = 0.054 μM for VBBPLA_2_ (Figure 2A); IC_50_ = 0.072 μM for VLPLA_2_ [18] and IC_50_ = 0.814 μM for NNOPLA_2_ (Figure 2B). 

In order to explore if the inhibitory effects of sPLA_2_s on platelet aggregation were related to their enzymatic activities, the native sPLA_2_s were treated by *p*-bromophenacylbromide (*p*-BPB) that modifies the histidine in the active center causing the inhibition of the catalytic activity. The *p*-BPB-treated enzymes were tested in the same conditions as the native vPLA_2_s. The treatment of all three svPLA_2_s by *p*-BPB resulted in complete loss of their catalytic activity that was accompanied by the loss of their inhibitory effect on collagen-induced platelet aggregation. 

### 2.3. Inhibitory Effect of Snake Venoms and Their sPLA_2_s on Bacteria

#### 2.3.1. Acute Toxicity to *Vibrio fischeri*

For the evaluation of the acute toxicity of studied enzyme preparations, naturally luminescent gram-negative bacteria *V. fischeri* were used. In these bacteria, the exposure to toxicants causes rapid decrease of their bioluminescence whereas the effect is dose-dependent [50]. In the current study, in addition to svPLA_2_s also the effect of the whole venom was evaluated. As a toxicity endpoint, inhibition of bacterial bioluminescence after 15 min of exposure to the whole venom or sPLA_2_s was used. In general, the venoms and sPLA_2_s were not acutely toxic to *V. fischeri*. Also, the sPLA_2_s were not acutely toxic: only enzyme from *V. lebetina* inhibited the luminescence of bacteria at <100 μg/mL (<7.31 μM) level, the 15-min EC_50 _was 58 μg/mL, *i.e.*, 4.24 μM; Table 1).

**Table 1:** Acute toxicity (15-min EC_50_, μg/mL) of venoms and sPLA_2_s from different snakes to bacteria *Vibrio fischeri*. As a toxicity endpoint, inhibition of the bacterial bioluminescence was used.

Tested item	Acute toxicity (15-min EC_50_, μg/mL)			
	3,5-DCP *	*V. b. berus*	*V. lebetina*	*N. n. oxiana*
Venom	3–4	370	944	>1315
PLA_2_	3–4	>909 (>65.76 μM)	58 (4.24 μM)	>606 (>45.81 μM)

* 3,5-dichlorophenol (a positive control).

#### 2.3.2. Inhibitory Effect of the Snake Venom PLA2s on Bacterial Growth

**Figure 3:**
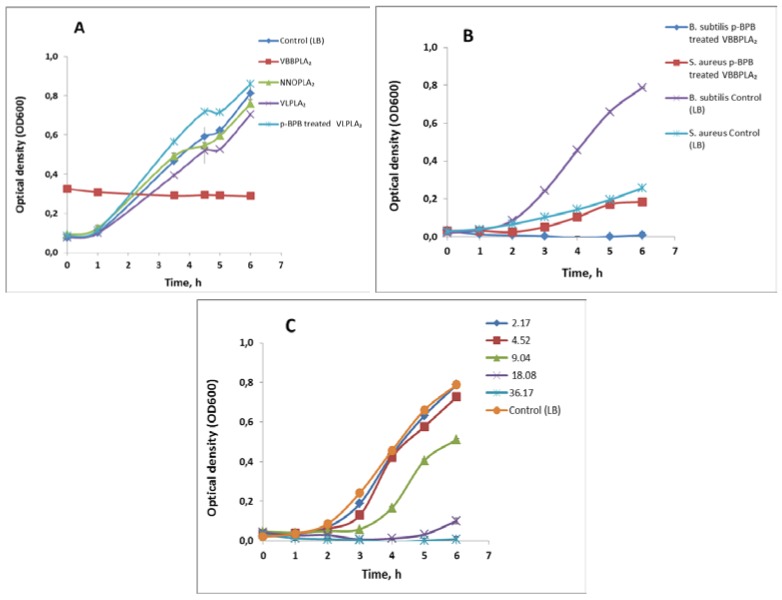
The effect of different snake venom sPLA_2_s on the growth of bacteria in LB medium at 30 °C. (**A**) The effect of different snake venom sPLA_2_s (500 μg/mL, *i.e.*, 36.2 μM for VBBPLA_2_; 37.8 μM for NNOPLA_2_; 36.5 μM for VLPLA_2_) on the growth of gram-positive bacteria *Bacillus subtilis* BR151. The different svPLA_2_s are indicated as data labels; (**B**) The effect of *p*-BPB-treated *V. berus berus* sPLA_2_ (500 μg/mL = 36.17 μM) on the growth of gram-positive bacteria *Bacillus subtilis* BR151 and *Staphylococcus aureus*. Growth of not treated bacteria is shown as data labels; (**C**) The effect of different concentrations of *p*-BPB-treated *V. berus berus* venom sPLA_2_ on the growth of *Bacillus subtilis* BR151; concentrations (μM) are shown as data labels. Results are reported as means ± SD (*n* = 3).

The inhibitory effect of svPLA_2_s on bacterial growth (a chronic toxicity) was evaluated at 500 μg/mL (36.2 μM for VBBPLA_2_; 37.8 μM for NNOPLA_2_; 36.5 μM for VLPLA_2_) level of the enzymes. The effect of VBBPLA_2_ on the growth of gram-positive bacterial strains was studied in parallel for the native enzymes and *p*-bromophenacylbromide-inactivated VBBPLA_2_s. The results are shown in Table 2 and Figure 3. Although the tested concentration was relatively high, none of the svPLA_2_s inhibited the growth of gram-negative bacteria *Escherichia coli* but there were inhibitory effects in case of some enzyme preparations on gram-positive bacterial strains (Figure 3A–C). Specifically, the *V. berus berus* PLA_2_ was most potent and totally (100%) inhibited the growth of *B. subtilis* (Figure 3A). The total growth inhibition of *B. subtilis* was also observed in case of *p*-BPB-inactivated VBBPLA_2 _(Figure 3B) whereas the effect was dose-dependent (Figure 3C). PLA_2_ from *V. lebetina* showed also some inhibitory effect (13%) towards *B. subtilis* but this inhibitory effect was not observed in case of *p*-BPB-inactivated enzyme (Figure 3A). Intact VBBPLA_2_ preparations (Table 2) had no inhibitory effect on gram-positive bacteria *S. aureus* but there was some inhibitory effect in case of inactivated enzyme (Figure 3B; Table 2). The *N. naja oxiana* PLA_2_ was inhibitory (42%) towards *S. aureus* (Table 2). 

**Table 2:** Inhibition of the bacterial growth (incubation time 6 h) in LB medium at 30 °C supplemented by svPLA_2_s (500 μg/mL, *i.e.*, 36.2 μM for VBBPLA_2_; 37.8 μM for NNOPLA_2_; 36.5 μM for VLPLA_2_) from three different snakes.

		Inhibition of the bacterial growth, % (*t* = 6 h)				
Bacteria(Gram staining)		svPLA_2_				
		*V. b. berus*	*V. b. berus* *	*V. lebetina*	*V. lebetina* *	*N. n. oxiana*
*Escherichia coli*	Gram (−)	No effect	not tested	No effect	No effect	No effect
*Bacillus subtilis*	Gram (+)	100% **	99% **	13%	No effect	Slight effect (6.5%)
*Staphylococcus aureus*	Gram (+)	No effect	29%	No effect	No effect	42%

* histidine in PLA_2_ was modified by *p*-bromophenacylbromide, to inactivate its catalytic activity; ** growth was inhibited by 100% but the viability of bacteria remained unchanged (*i.e.*, after the 6 h exposure to enzyme preparation, bacteria were able to grow on agarized LB-medium; data not shown).

### 2.4. Effects of Snake Venom PLA_2_s on Cancer Cells Viability

**Figure 4:**
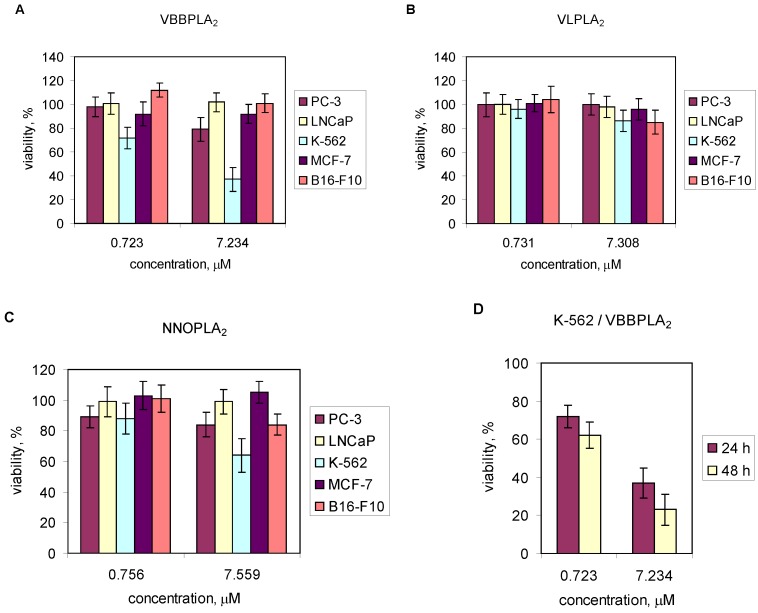
Effect of svPLA_2_s on viability of PC-3, LNCaP, K-562, MCF-7 and B16-F10 cells *in vitro*. (**A**–**C**) Cells were seeded in 96-well plates at a density 10^5^ cells/mL and incubated at 37 °C for at least 24 h. After treatment with snake venom PLA_2_s (~0.7 and ~7 μM) for 24 h, the viability of the cells was determined by MTT assay (PC-3) or by water-soluble tetrazolium salt WST-1 assay (LNCaP, K-562, MCF-7 and B16-F10); (**D**) K-562 cells were treated with VBBPLA_2_ (0.72 μM and 7.23μM) for 24 and 48 h. Data are means (±SD) from two independent experiments performed in triplicate.

Cancer cell lines (PC-3, LNCaP, MCF-7, B10-F16 and K-562) were exposed to PLA_2_s from *V. lebetina*, *V. berus berus* and *N. naja oxiana* at concentrations of 10 and 100 μg/mL (~0.7 and ~7 μM). The results are shown in Figure 4. There was no inhibitory effect of studied PLA_2_ preparations towards LNCaP cells in this concentration range (Figure 4A–C). The viability of PC-3 cells was not changed after treating with 7.31 μM of VLPLA_2_ (Figure 4B). NNOPLA_2_ had no cytotoxic effect on MCF-7 cells (Figure 4C), VBBPLA_2_ and VLPLA_2_ only slightly reduced the viability of MCF-7 cells (Figure 4A,B). VLPLA_2 _and NNOPLA_2_ decreased viability of B16-F10 cells about 17% (Figure 4B,C), VBBPLA_2 _had no effect (Figure 4A). All three enzymes inhibited the viability of K-562 cells (Figure 4A–C), although VLPLA_2_ had only slight effect (Figure 4B). The most potent inhibitory effect was observed in case of VBBPLA_2_. After 48 h treatment of K-562 cells with 7.23 μM of VBBPLA_2_, the cellular viability reduced to 20% (Figure 4D). *p*-BPB-treated VBBPLA_2_ inhibited the viability of K-562 cells by 27%. VBBPLA_2_ reduced the viability of K-562 cells in time- and dose-dependent manner. 

**Figure 5:**
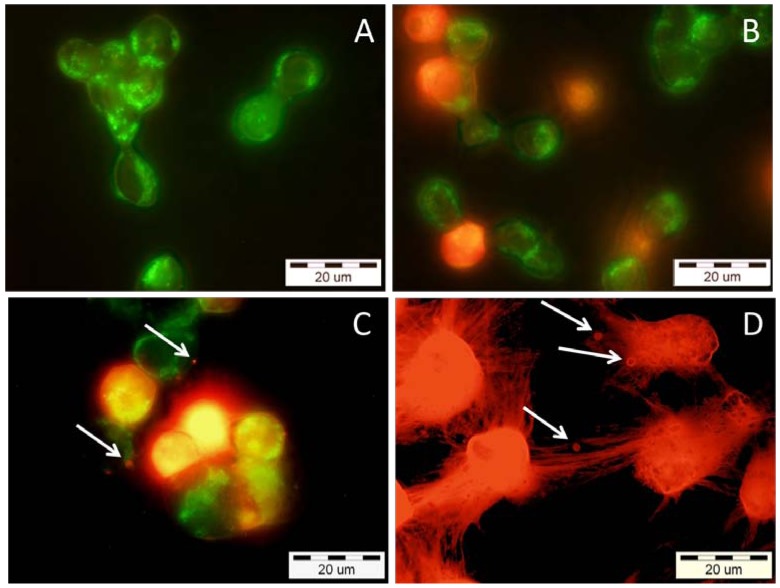
Epifluorescence micrographs of human K-562 cells after incubation with different concentrations of VBBPLA_2_. After the exposure, the cells were stained with both Annexin V-FITC and propidium iodide, to visualize the early and late stage of apoptosis and/or necrosis of the cells, respectively. (**A**) 0.36 μM (expose 24 h)—early apoptotic cells with intact membranes—green; (**B**) 0.36 μM (expose 28 h)—mixture of early apoptotic cells (green) and cells which have already lost their membrane integrity (orange); (**C**) 0.72 μM (expose 24 h)—late apoptotic cells (orange to red) with blebbes (white arrows) and green membrane fragments; (**D**) 7.23 μM (expose 24 h)—totally destroyed necrotic (red) cells with membrane blebbes (white arrows).

To evaluate whether the cytotoxicity effect of VBBPLA_2_ on K-562 cells (Figure 4D) was necrotic or apoptotic, the treated cells were stained with Annexin-V-FITC and propidium iodide (PI) (Figure 5). One characteristic feature of apoptosis is the externalisation of the lipid phosphatidyl serine (PS) from the inner to the outer plasma membrane. Annexin-V is a calcium-dependent phospholipid-binding protein that specifically binds PS and hence stains apoptotic cells. When used in conjunction with a live/dead cell discriminator such as propidium iodide, which measures membrane integrity, the bright green early apoptotic cells (Annexin-V positive) can be distinguished from the red colored late apoptotic/necrotic cells (PI positive). PI stains the cells with ruptured plasma membrane as cells with intact membranes are not permeable to PI. Thus, PI stains both, the cells in the late stage of apoptosis and the cells in necrosis. The treatment of K-562 cells with 0.36 μM VBBPLA_2_ caused the loss of cell membrane’s asymmetry which is a sign of early apoptosis (Figure 5A). 

The transition from apoptosis to necrosis is a loosely defined continuum that necessitates recognition of the various stages of the process. Therefore, we performed a time course experiment (the cells were photographed after 24 h and 28 h of incubation) to prove that the cells were traversing through early apoptosis before reaching the late apoptosis/necrosis (Figure 5A,B). In our study the bright green cells (Annexin-V positive early apoptotic cells) turned to orange (Annexin-V and PI positive late apoptotic cells) when VBBPLA_2_ concentration was increased from 0.36 μM (Figure 5A) to 0.72 μM (Figure 5C) but also in case of lower VBBPLA_2_ concentration (0.36 μM) if the incubation time was prolonged to 28 h (Figure 5B). The cells treated with 7.23 μM VBBPLA_2_ appeared totally destroyed, but it was still possible to detect the characteristic sign of apoptosis—membrane blebbing (Figure 5D, white arrows).

## 3. Discussion

Snake venom sPLA_2_s exhibit a large variety of pharmacological effects. In this work we compared the effects of sPLA_2_s originating from the venoms of three different snakes on human platelets, different bacteria and five types of cancer cells *in vitro. Naja naja oxiana* PLA_2_ belongs to PLA_2_ from old world snakes (group I) and has different disulfide bond pattern than PLA_2_s from new world’s snakes such as VBBPLA_2_ and VLPLA_2_ (group II).

Kini and Evans [15] divided snake venom PLA_2_s based on their effects on platelet function into three classes: class A involves PLA_2_s which initiate platelet aggregation, class B PLA_2_s cause only the inhibition of platelet aggregation induced by several physiological agonists such as collagen and class C involves PLA_2_s that have dual activity acting as inducer and inhibitor, depending of conditions. Classes B and C are both subdivided into two subgroups. Inhibitory activity of class B1 PLA_2_s (but not class B2) is dependent on their catalytic activity. Results of the current study show that VBBPLA_2 _and NNOPLA_2_ belong to class B1. In class B1 the inhibitory effects against platelets aggregation have been explained by hydrolysis of phospholipids from the plasma and/or from lipoproteins and the formation of lysophosphatidylcholine (lysoPC) [21,22,51]. The platelet aggregation inhibitory effects of PLA_2_s have shown to be dependent on plasma factor for several snake venom PLA_2_s, including VLPLA_2_ [18], the antiplatelet PLA_2_ purified from the venoms of *Austrelaps superba* [51], *Lachesis muta* [21,52], and *Micropechis ikaheka* [53]. Yuan *et al.* [51] showed that the formation of lysoPC after incubation with snake venom PLA_2_ correlated with the inhibition of platelet aggregation. 

The isoelectric point values of snake venom PLA_2_s vary and therefore PLA_2_s are classified as acidic, neutral or basic. This property may affect the binding affinity and specificity of PLA_2_s to phospholipid membranes. However, pI values of PLA_2_s are not predictive for their effect on platelet aggregation: the acidic VLPLA_2 _ [18], acidic PLA_2_s from the venoms of *Trimeresurus gramineus* [13] and *Agkistrodon acutus* [14] and basic PLA_2_s from *V. berus berus* venom (this work), from *Acanthopis praelongus* venom [16] and acanthins from *Acanthopis antarcticus* venom [22] are all potent platelet inhibitors. On the contrary, bothropstoxin-II (Bthtx-II), a basic Asp^49^ phospholipase A_2_ isolated from *Bothrops jararacussu* snake venom was able to induce platelet aggregation in a concentration-dependent manner [17]. NNOPLA_2 _with almost neutral pI (6.7) inhibited collagen induced platelet aggregation more slowly than VBBPLA_2_ and VLPLA_2_ (Figure 2).

Although only PLA_2_ from *V. lebetina* but not the PLA_2_s from *V. berus berus* and *N. naja oxiana* showed acute toxic effect on *Vibrio fischeri* at 4.24 μM level (Table 1), many snake venom phospholipases A_2_ have been shown antibacterial and antiparasitic properties. For example, the Lys^49 ^protein from *Bothrops asper* venom showed bactericidal activity on both, gram-positive and gram-negative bacteria [27]. Contrarily, the Lys^49^ BmarPLA_2_ from *Bothrops marajoensis* showed no antibacterial and antiparasitic effects [36]. Two myotoxic Asp^49^ PLA_2_s from *Bothrops neuwiedi pauloensis* venom were bactericidal towards *Escherichia coli* and *Staphylococcus aureus* [31]. Myotoxin I Lys^49^ PLA_2_ from *Bothrops atrox* venom was weakly bactericidal against *E. coli* [30]. Myotoxin I Lys^49^ PLA_2_ and myotoxin II Asp^49^ PLA_2_from *Bothrops jararacussu* venom showed antibacterial effect against gram-negative bacteria *Xanthomonas* [54]. Myotoxic Asp^49^ PLA_2_ MTX-I and Lys^49^ PLA_2_ MTX-II isolated from *Botrops brazili* venom and cationic synthetic peptides derived from their 115–129 *C*-terminal region displayed toxic effects against *E. coli*, *Candida albicans* and *Leishmania sp*. and human T-cell leukemia (JURKAT) cell lines [55]. 

In the current study, the 36.17 μM VBBPLA_2_ totally inhibited the growth of gram-positive bacteria *Bacillus subtilis* (Table 2, Figure 3A) but did not inhibit the growth of other bacterial strains analyzed (Table 2). VBBPLA_2 _has highly cationic nature as it contains numerous positively charged Arg and Lys residues that may promote its binding to negatively-charged outer surface of bacteria. The majority of antimicrobial peptides are positively charged at physiological pH, and prevailing view is that their selectivity stems from electrostatic attraction of the cationic peptide to the anionic bacterial membranes [56]. However, to another gram-positive bacterium, *Staphylococcus aureus*, native VBBPLA_2 _had no inhibitory effect (Table 2).

The activity and expression of several PLA_2 _isoforms are increased in several human cancers, including breast, pancreatic and prostate cancers, suggesting that these enzymes may have a central role in both tumor development and progression and thus can be targets for anticancer drugs [12,57]. On the other hand, some snake venom PLA_2_s may have antitumoral activity [12]. Crotoxin, a noncovalent complex (formed by two nonidentical subunits: a basic PLA_2 _crotoxinB and a nonenzymatic acidic crotoxinA) isolated from the venom of *Crotalus durissus terrificus,* exhibits a preferential cytotoxic activity against various types of tumor cells including K-562 cells [58], MCF-7 cells [59] and lung adenocarcinoma A549 cells. Treatment of A549 cells with crotoxin significantly inhibited the cell growth in a dose-dependent manner and displayed anti-angiogenic effects *in vitro* [60]. Crotoxin has been used in the treatment of different advanced carcinomas [61]. It has been shown that *bl*D- PLA_2_ from *Bothrops leucurus* snake venom reduced K-562 cellular viability in a dose-dependent manner causing disruption of cellular membrane integrity [62]. Several secreted PLA_2_s were found to play role in apoptosis [63]. PLA_2 _from *Naja naja atra* venom induced apoptotic cell death of K-562 cells [43]. A Lys^49^ phospholipase A_2_ from *Protobothrops flavoviridis* venom induced caspase-independent apoptotic cell death accompanied by rapid plasma-membrane rupture in human leukaemia cells. However, Asp^49^ PLA_2_ from the same venom failed to induce death of JURKAT cells [1]. 

In this study, different cancer cell lines (PC-3, LNCaP, K-562, MCF-7, B10-F16) were exposed to different PLA_2_s from *V. lebetina*, *V. berus berus* and *N. naja oxiana*. At the highest concentration tested (~7 μM), there was no inhibitory effect of studied PLA_2_ preparations towards LNCaP cells (Figure 4A–C). This is coherent with the data of Sved *et al.* [64] on the consistent and dose-dependent stimulatory effect of human recombinant sPLA_2_-IIA on LNCaP cell growth. In the current study, the most potent inhibitory effect of studied svPLA_2_s was observed for VBBPLA_2 _towards human chronic myeloid leukemic cell line K-562 (Figure 4D). In addition, *p*-BPB-treated inactive VBBPLA_2_ yielded 27% loss of viability in K-562 cells. Thus, VBBPLA_2_-induced cell death is dependent not only of enzymatic activity.

## 4. Materials and Methods

### 4.1. Materials

The venoms of *V. lebetina* and *N. n. oxiana* were commercial preparations from Tashkent Integrated Zoo Plant (Uzbekistan), *V. b. berus* venom was obtained from Khimki Serpentarium (Moscow, Russia). Sephadex G-100 (superfine) was product of Pharmacia (Uppsala, Sweden). 2,5-dihydroxybenzoic acid (DHB), 3,5-dichlorophenol, bovine serum albumin (BSA), ovalbumin, carboanhydrase, 3-(4,5-dimethylthiazol-2-yl)-2,5-diphenyltetrazolium bromide (MTT), soybean trypsin inhibitor, Substance P, Cytochrome C, insulin B chain, *p*-bromophenacylbromide (*p*-BPB) and camptothecin were from Sigma (St. Louis, MO, USA), trypsin (Promega, Madison, WI, USA). WST-1 was from Roche Diagnostics, collagen from Chronolog. Annexin V/Dead Cell Apoptosis Kit with FITC annexin V and propidium iodide (PI) were from Invitrogen, Eugene, OR, USA. All other reagents used were of analytical grade.

### 4.2. Purification of Enzymes

*Vipera lebetina* PLA_2_ was purified according to Vija *et al.* [18], *Vipera berus berus* PLA_2_ (VBBPLA_2_) was separated from the venom as described by Križaj *et al* [48]. *Naja naja oxiana* venom PLA_2_ (NNOPLA_2_) was purified by gel filtration on Sephadex G-50 sf. and hydrophobic chromatography on pentylagarose. Purity and molecular masses of enzymes were detected by SDS-PAGE and MALDI-TOF MS (see 4.6.). 

### 4.3. PLA_2_ Assay

Phospholipase A_2_ activity was assayed by titrimetric method using egg yolk phosphatidylcholine as a substrate [65]. Briefly, one egg yolk was added to 100 mL of bidistilled water and aqueous emulsion was prepared by homogenisation. Per assay, 1.5 mL of the egg yolk emulsion was diluted with 3 mL of Triton X-100 and CaCl_2 _being 0.75% and 0.15 mM, respectively. The pH was set at 8.0; 10 μL (0.1 mg/mL) of PLA_2 _sample was added and the fatty acids released were titrated with 10 mM KOH using a pH-stat (TTT80/pHM84/ABU80, Radiometer, Copenhagen, Denmark) at 25 °C. 

### 4.4. PLA_2_ Activity Inhibition with p-bromophenacylbromide (p-BPB)

PLA_2_s (0.4 mg) were dissolved in 0.4 mL of 0.1 M ammonium acetate (pH 7.4) containing 0.4 mM of *p*-BPB and incubated for 24 h at room temperature. Excess of the reagents was removed by ultrafiltration through the microspin filter (cut-off 5000 MW, Cole-Parmer, Vernon Hills, IL, USA), the protein fraction was washed with 0.1 M ammonium acetate (pH 7.4) and lyophilized. 

### 4.5. Protein Quantification

Protein concentrations were determined using the Pierce micro BCA kit. Bovine serum albumin was used as a standard. During the process of column chromatography, the elution profile of proteins was followed by the absorbance at 280 nm.

### 4.6. Molecular Mass Detection and Isoelectric Focusing of Proteins

The molecular masses of the purified proteins were determined by SDS-PAGE on 12.5% polyacrylamide gels using the method of Laemmli [66]. Molecular mass standards for SDS-PAGE were albumin—66 kDa, ovalbumin—45 kDa, carboanhydrase—29 kDa, soybean trypsin inhibitor—20 kDa, cytochrome C—12.3 kDa. 

The molecular masses of the fractions were also determined using a home-built matrix-assisted laser desorption/ionization-time of flight mass spectrometer (MALDI-TOF MS) (National Institute of Chemical Physics and Biophysics, Tallinn, Estonia). Before the analysis the freeze-dried samples of protein fractions were dissolved in 5 μL of 50% acetonitrile containing 0.1% trifluoroacetic acid. Aliquots of 0.5 μL were applied onto the target, allowed to air dry and 0.5 μL of the matrix solution (2,5-dihydroxybenzoic acid) was applied to the target and allowed to dry in air. The mass calibration standards were cytochrome C, insulin B chain. A nitrogen 337 nm laser (4 ns pulse) was used and at least 30–40 shots were summarized.

Analytical isoelectric focusing was performed on 5% polyacrylamide gel plates according to the method of Vesterberg [67] in Multiphor 2117 (LKB, Bromma, Sweden) apparatus in the pH range of 3.6–9.3. Isoelectric focusing markers were amyloglucosidase (pI 3.60), soybean trypsin inhibitor (pI 4.55), β-lactoglobulin A (pI 5.20), bovine carbonic anhydrase B (pI 5.85), human carbonic anhydrase B (pI 6.55), horse myoglobin-acidic band (pI 6.85), horse myoglobin-basic band (pI 7.35) lentil lectin-acidic band (pI 8.15), lentil lectin-middle band (pI 8.45), lentil lectin-basic band (pI 8.65) and trypsinogen (pI 9.30). The gels were stained for proteins with Coomassie Brilliant Blue R250.

### 4.7. In-Gel Tryptic Digestion and Mass Fingerprinting of Proteins

After visualization with Coomassie Blue the gel-electrophoresis bands of protein in interest (native or reduced) were excised from SDS-PAGE gels, each gel slice cut into small pieces (1 mm^2^), placed into eppendorf tubes and treated as described earlier [68]. Equal volumes (0.5 μL) of the peptide mixture and the matrix (2,5-dihydroxybenzoic acid, or α-cyano-4-hydroxycinnamic acid) were mixed on the MALDI-TOF plate. The mass calibration standards were substance P and angiotensin II. 

### 4.8. Preparation of Human Platelet Suspension and Collagen-Induced Platelet Aggregation Assay

Collagen-induced platelet aggregation assays were performed in human platelet-rich plasma (PRP). Blood was collected from healthy adult volunteers who had not taken any medication for at least two weeks prior to sampling. The blood was collected according to the permissions LO2354 (14.12.2010) and LO2513 (21.07.2011).

In order to obtain PRP the blood was dispensed into polystyrene tubes containing 0.129 M sodium citrate (9:1 *v*/*v*) as anticoagulant and after centrifugation at 180 × *g* at room temperature for 10 min platelet suspensions were prepared according to the previously described protocol [69]. Platelet aggregation was measured photometrically in a Whole-Blood aggregometer (Chronolog Corporation, Havertown, PA, USA) under continuous stirring at 900 rpm at 37 °C. Control experiments were done using collagen (platelet agonist) alone. 

### 4.9. Antibacterial Activity

#### 4.9.1. Bacterial Strains

Altogether, four different bacterial strains were used. Naturally luminescent *Vibrio fischeri* NRRL-B-11177 was purchased from Aboatox (Turku, Finland). Constitutively luminescent *Escherichia coli* MC1061(pSLlux) and *Staphylococcus aureus* RN4220(p602/22lux) were constructed earlier by Ivask *et al.* [70]. *Bacillus subtilis* BR151 was obtained from Turku University (Finland). Two former strains are gram-negative and two latter ones gram-positive bacteria. 

#### 4.9.2. Analysis of Antibacterial Activity of PLA_2_s

Antibacterial activity of sPLA_2_s was analyzed using two different methods: (i) inhibition of the luminescence of naturally luminescent gram-negative bacterium *Vibrio fischeri* after 15 minutes of exposure and (ii) inhibition of the growth of gram-negative bacteria *Escherichia coli* and *Staphylococcus aureus* and gram-positive bacteria *Bacillus subtilis* upon 6 hour exposure to PLA_2_s of various snakes.

##### 4.9.2.1. Bioluminescence Inhibition Assay Using *Vibrio fischeri*

The *Vibrio fischeri* test bacteria were prepared as described in Kurvet *et al.* [71]. Briefly, *V. fischeri* bacterial suspension was obtained by rehydration of freeze-dried *V. fischeri* Reagent (Aboatox, Turku, Finland) using 2% NaCl, stabilized for 40 min at 4 °C and then at 20 °C for 40 min and then used for testing. 2% NaCl served as a test diluent and as a negative control. 3,5-dichlorophenol was used as a positive control. The assay was performed at 20 °C instead of 15 °C recommended by standard operational procedure of Microtox^™^ (AZUR Environmental, Carlsbad, CA, USA) as most luminometers do not allow the temperature adjustment to 15 °C.

Testing was performed essentially as described in Kahru [50] using 1253 Luminometer and respective software for the data reduction (both BioOrbit, Turku, Finland). Toxicity (15-min EC_50_), *i.e.*, the concentration of svPLA_2 _causing a 50% reduction in light output of bacteria after 15-min contact time, was determined from respective concentration-effect curves. 

##### 4.9.2.2. Bacterial Growth Inhibition Assays

*E. coli*, *S. aureus* and *B. subtilis* were maintained in LB agar plates (LabM, Lancashire, UK) supplemented with respective antibiotics (see below) at +4 °C. For the toxicity tests, bacteria were cultivated (on a shaker at 200 rpm, 30 °C) overnight in 3 mL of LB medium. As a test medium for the growth inhibition assays and as a diluent for svPLA_2_s LB medium without NaCl was used. Ampicillin (100 μg/mL) in case of *E. coli* and kanamycin (50 μg/mL) in case of *S. aureus* were added to LB medium. No antibiotics were added to *B. subtilis* culture medium. For the assay, overnight bacterial culture was diluted 1:25 in LB medium containing respective antibiotics (see above). Then, 100 μL of test bacteria was added to 100 μL of the svPLA_2_ dilution. Each svPLA_2_ was tested in following concentrations: 500, 250, 125, 62.5 and 31.25 μg/mL. Each svPLA_2 _concentration was tested in three and the controls in ten replicates. 96-well polystyrene microplates with transparent bottoms and not-transparent sides of the wells (Greiner Bio-One, Frickenhausen, Germany) were used. Optical density of the bacterial suspensions at 600 nm (OD_600_) was measured using Multiscan Spectrum spectrophotometer (Thermo Scientific, Vantaa, Finland). The measurements were performed in 1 h intervals till 6 h and then also 24 h data were registered. Between the measurements till 6 h the plates were incubated at 30 °C on a shaker (Heidolph Titramax 1000, Schwabach, Germany) at 750 rpm and then statically overnight in the incubator at 30 °C. The inhibition of the growth of bacteria was calculated as percentage of the non-exposed control. 

To evaluate the ability of svPLA_2-_exposed bacteria (after 6 h and 24 h incubation) to grow on solid media, 1 μL of bacterial suspension was streaked onto Petri dishes with LB agar containing no antibiotics. The growth of bacteria was visually checked after incubation of Petri plates at 30 °C for 48 h. 

### 4.10. Human Cell Lines and Toxicity Testing of sPLA_2_s

The human prostate cancer cell lines PC-3, LNCaP, human chronic myeloid leukemic cell line K-562, breast cancer cell line MCF-7 and mouse melanoma cell line B16-F10 were purchased from the American Type Culture Collection (ATCC; Manassas, VA, USA). PC-3 cells were maintained in DMEM/F-12 medium (Gibco, Grand Island, NY, USA), LNCaP, K-562, MCF-7 and B16-F10 cells in RPMI 1640 medium (Gibco, Grand Island, NY, USA), supplemented with 10% fetal bovine serum (Gibco) and antibiotics (100 units/mL penicillin and 100 μg/mL streptomycin) at 37 °C and 5% CO_2_ in a fully humidified atmosphere.

#### 4.10.1. Analysis of the Viability of the Cells

The viability was determined by the MTT assay (PC-3 cells) and WST-1 assay (LNCaP, K-562, MCF-7 and B16-F10) based on the reduction of MTT or WST-1 by viable cells, respectively.

##### 4.10.1.1. MTT Assay

Human prostate cancer PC-3 cells were seeded in 96-well plates (Sarstedt, Germany) at a density of 1–2 × 10^5^ cells/ml. After 24 h of incubation 37 °C the cells were incubated with svPLA_2_s diluted with medium and added to the wells at final concentrations of 10 and 100 μg/mL. The cells not treated with sPLA_2_ served as a control. After certain time intervals, MTT solution was added to each well at a final concentration of 0.5 mg/mL and the plates were incubated at 37 °C for 4 h. The MTT formazan product was dissolved by addition of 110 μL acidified 2-propanol (in 0.04 N HCl) to each well. The absorbance was detected in micro-plate reader (Multiskan Spectrum, Thermo, Vantaa, Finland) at 540 nm. Cell survival rate was calculated as (absorbance of the treated wells)/(absorbance of the control wells) × 100%. 

##### 4.10.1.2. WST-1 Assay

Human LNCaP, K-562, MCF-7 and B16-F10 cells were seeded in 96-well plates at a density of 1–2 × 10^5^ cells/ml. After 24 h of growth cells were incubated with svPLA_2_s diluted with medium and added to the wells at the desired final concentrations (10 and 100 μg/mL). The cells that were not treated with protein served as control cells. After various time intervals 10 μL/well WST-1 solution was added to each well and the plates were incubated for 1–2 h at 37 °C and 5% CO_2_. The absorbance of the WST-1 formazan salt was detected in micro-plate reader at 450 nm. Cell survival rate was calculated as (absorbance of the treated wells)/(absorbance of the control wells) × 100%.

#### 4.10.2. Apoptosis Detection Using Annexin V-FITC and Propidium Iodide (PI)

The detection of K-562 cells apoptosis was performed according to the instructions of FITC Annexin-V/Dead Cell Apoptosis Kit with FITC Annexin-V and PI (Invitrogen, Eugene, OR, USA). The suspension of K-562 cells was seeded into 24-well plates (2 × 10^5^ cells/well) on round cover slips and incubated at 37 °C with 5% CO_2_ for 24 h. After this period, the cells were treated with VBBPLA_2_ (0.36, 0.72 and 7.23 μM) for 24 h. In case of 0.36 μM the treatment was prolonged to up to 28 h. 4 μM camptothecin-treated cells (4 h) were used as a positive control for apoptosis. The cells were washed twice with cold phosphate-buffered saline (PBS) and 200 μL of Annexin-V binding buffer, 10 μL of Annexin-V-FITC and 10 μL of PI working solution were added. After incubation in the dark for 15 min at room temperature the reaction mixture was removed and the cells were washed with Annexin-V binding buffer. Then, the cover slips with cells were taken out from the wells and the mounted preparations were made. The viability of the treated and non-treated (control) K-562 cells was observed under an epifluorescence microscope Olympus CX41 with a 100× oil immersion objective lens and fluorescence optics (excitation at 488 nm, >515 nm emission for Annexin V-FITC (green) and for propidium iodide (red)). The pictures were taken using an Olympus U-CMAD3 real time colour digital DP71 camera (Tokyo, Japan) using the CellB Software (Olympus Soft Imaging Solutions GmbH, Münster, Germany).

## 5. Conclusions

The adverse effects of PLA_2_s from *Vipera lebetina*, *Vipera berus berus* and *Naja naja oxiana* venom depended on venom (snake) as well as on target cells (platelets, different cancer cell types and bacteria). As a rule, the observed biological effects on platelets were observed already at 1 μg/mL level (<0.1 μM) and all three PLA_2_s were dose-dependently inhibiting the collagen-induced platelet aggregation. The chemical modification of histidine in studied PLA_2_s by *p*-bromophenacylbromide resulted in complete loss of their catalytic activity and inhibitory action on collagen-induced platelet aggregation. VBBPLA_2 _(but not the PLA_2_s from *V. lebetina* and *N. naja oxiana*) was totally inhibiting the growth of gram-positive *Bacillus subtilis* at 500 μg/mL (36.2 μM) whereas the inhibitory effect was not due to its catalytic activity but to other properties of the protein. To another gram-positive bacterium, *S. aureus*, native sPLA_2_ from *N. naja oxiana* inhibited the growth of bacteria by 42% but caused only slight inhibition of growth of *B. subtilis*. None of the studied svPLA_2_s was inhibitory to the growth of gram-negative bacteria *E. coli* even at 500 μg/mL (~37 μM) level.

The viability of the most sensitive cancer cell type (K-562) was reduced upon exposure of the cells to 7.2 μM VBBPLA_2_ and to some extent also by PLA_2_s from *V. lebetina* and *N. naja oxiana*. There was no inhibitory effect of all studied svPLA_2_ preparations towards LNCaP cells and low inhibitory effect (8%–20%) towards the PC-3, MCF-7 and B10-F16 cells. Thus, from the current suite of studied svPLA_2_s and test cells, VBBPLA_2_ was most growth inhibitory towards gram positive bacteria *B. subtilis* and K-562 cells *in vitro*.

## Supplementary Files

Supplementary File 1 Supplementary Information (PDF, 124 KB)
