# Supplementary material for: Interactions of PLA2-s from Vipera lebetina, Vipera berus berus and Naja naja oxiana Venom with Platelets, Bacterial and Cancer Cells
Source: Toxins (Basel). 2013 Jan 24;5(2):203–23. doi: 10.3390/toxins5020203 (PMC3640532; doi:10.3390/toxins5020203)
Supplement: Supplementary File 1 — Supplementary Information (PDF, 124 KB) [file toxins-05-00203-s001.pdf]

Supplementary Information

Figure S1. MALDI-TOF analysis of tryptic peptides derived from NNOPLA$_2$. The molecular masses and sequence fragments are marked in the figure.

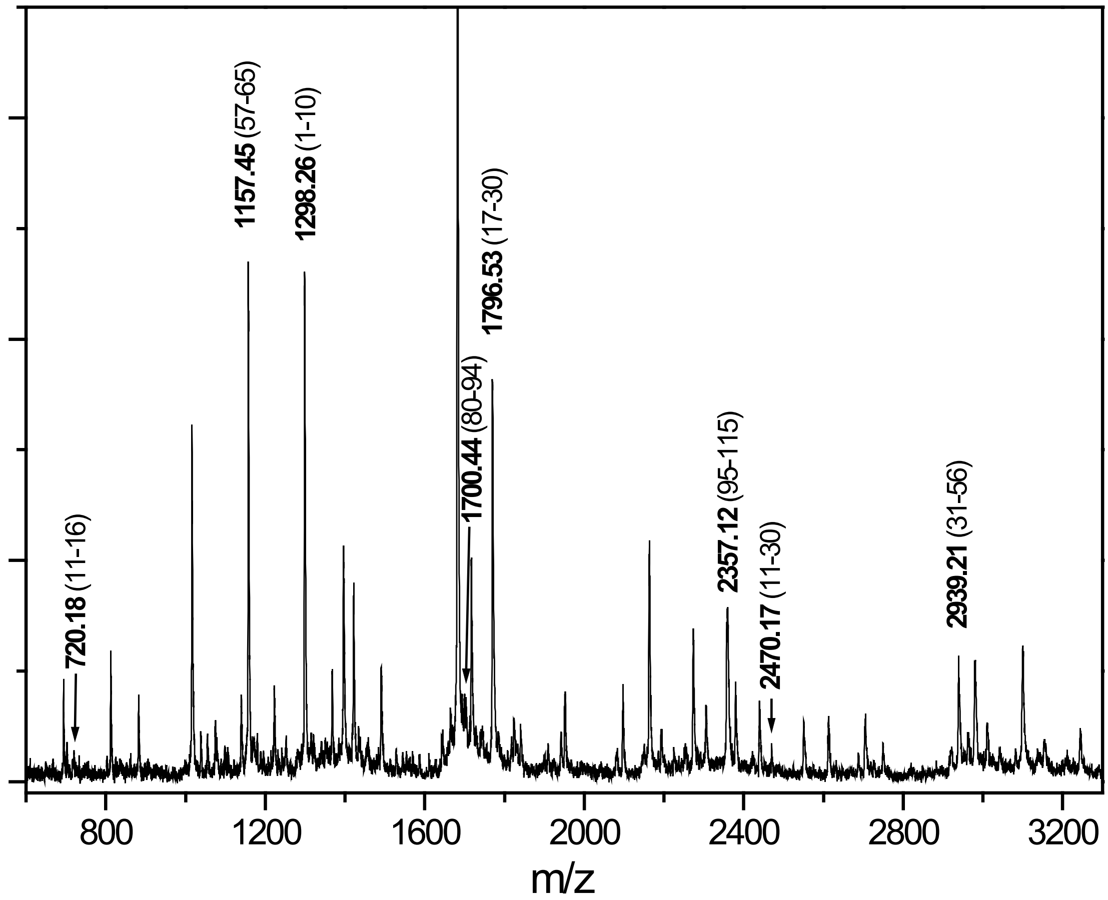

© 2013 by the authors; licensee MDPI, Basel, Switzerland. This article is an open access article distributed under the terms and conditions of the Creative Commons Attribution license (http://creativecommons.org/licenses/by/3.0/).
